# Genome-wide analysis of long-term evolutionary domestication in *Drosophila melanogaster*

**DOI:** 10.1038/srep39281

**Published:** 2016-12-22

**Authors:** Mark A. Phillips, Anthony D. Long, Zachary S. Greenspan, Lee F. Greer, Molly K. Burke, Bryant Villeponteau, Kennedy C. Matsagas, Cristina L. Rizza, Laurence D. Mueller, Michael R. Rose

**Affiliations:** 1 University of California, Irvine, Department of Ecology and Evolutionary Biology, Irvine, CA, 92617, USA; 2 Genescient Inc., Fountain Valley, CA 92708, USA; 3 Oregon State University, Department of Integrative Biology, Corvallis, OR 97331, USA.

## Abstract

Experimental evolutionary genomics now allows biologists to test fundamental theories concerning the genetic basis of adaptation. We have conducted one of the longest laboratory evolution experiments with any sexually-reproducing metazoan, *Drosophila melanogaster*. We used next-generation resequencing data from this experiment to examine genome-wide patterns of genetic variation over an evolutionary time-scale that approaches 1,000 generations. We also compared measures of variation within and differentiation between our populations to simulations based on a variety of evolutionary scenarios. Our analysis yielded no clear evidence of hard selective sweeps, whereby natural selection acts to increase the frequency of a newly-arising mutation in a population until it becomes fixed. We do find evidence for selection acting on standing genetic variation, as independent replicate populations exhibit similar population-genetic dynamics, without obvious fixation of candidate alleles under selection. A hidden-Markov model test for selection also found widespread evidence for selection. We found more genetic variation genome-wide, and less differentiation between replicate populations genome-wide, than arose in any of our simulated evolutionary scenarios.

The genetic basis of adaptation has historically been a major point of contention among evolutionary biologists^1^. In recent years, combining genome-wide sequencing and experimental evolution has emerged as a powerful method for parsing the genetic underpinnings of adaptation^1^,^2^,^3^,^4^. Termed the “evolve and resequence” (E&R) approach, these experiments involve sequencing laboratory populations that have been exposed to clearly defined selective pressures in the hopes of making direct connections between patterns of genotypic and phenotypic change^5^. In the case of largely or wholly asexual populations, genome-wide sequencing has been performed on clones derived from single individuals after many generations of adaption to novel conditions^3^,^6^. Since such asexual populations are expected to undergo successive rounds of selective sweeps that purge genetic variation genome-wide^7^, this is a reasonable approach to the characterization of chiefly clonal evolutionary processes.

In the case of outbreeding sexual species, such as *Drosophila melanogaster,* the more common sequencing strategy in E&R experiments has been to pool multiple individuals within or across evolving replicated laboratory populations^1^,^8^. This is often referred to as the “pool-seq” approach^4^. Results from E&R experiments in outbreeding sexual species using this pool-seq approach have revealed abundant genetic variation genome-wide, and suggest that adaptation is primarily due to selection on standing genetic variation^4^. However, as most of those studies typically feature populations with relatively small effective population sizes that have been subjected to only a few dozen generations of selection, there is a limit to the conclusions that can be drawn from them regarding the relative importance of selective sweeps versus selection acting on standing genetic variation^9^,^10^,^11^, particularly as selective sweeps are likely to take much longer than the few dozens of generations commonly used in selection experiments with metazoa, as opposed to experimental evolution with microbes^6^.

Genome-wide sequencing of genetic variation present in experimentally evolving sexual populations after many generations of selection remains of interest as a method for addressing the relative importance of selective sweeps, particularly from the standpoint of alleles being driven to fixation. Hitchhiking effects arising from successive selective sweeps are not expected to purge genetic variation genome-wide in sexual populations immediately^7^,^12^. But sufficiently many such selective sweeps acting in conjunction with reductions in heterozygosity resulting from background selection and genetic drift conceivably could progressively purge genetic variation, given the moderately small population sizes commonly used in experimental evolution with sexual species^13^. The data analyzed by Burke *et al*.^1^ do not show a widespread purging of genetic variation in populations that had evolved in the lab for some decades. However, save for a single replicate sequenced individually, this study featured data generated from pooling across replicates, which could have potentially masked genetic fixation in individual replicate populations. This raises the question whether genome-wide sequencing of independently evolving, replicate, sexual populations that have been maintained in the lab for hundreds of generations will indeed show a pattern of generally purged genetic variation when these populations are resequenced separately.

Here we show that such long-evolved moderate-sized sexual populations do not exhibit the general lack of genetic variation that is characteristic of long-evolved clonal populations. Instead, after sequencing five independent replicate populations sharing a common selection regime, and more than 900 generations of sustained directional selection, we find widespread maintenance of genetic variation genome-wide. While it could be argued we still do not have sufficient generations of selection or population sizes large enough, our study features the best data collected from laboratory evolution to date with respect to the long-term evolution of patterns of genetic variation in sexually reproducing populations of multicellular eukaryotes. Furthermore, we compare measures of variation within and differentiation between our populations to simulated data from a number of evolutionary scenarios. We consistently find that there is more variation maintained in our populations, and less differentiation between replicate populations, than is found in any of the evolutionary scenarios we simulated. Lastly, we look at patterns of genetic variation and the frequency distribution of genetic variation to test for selective sweeps.

## Materials and Methods

### Experimental material

The novel experimental material analyzed here is pooled genomic DNA obtained from each of the five “B” populations maintained in the Rose laboratory^14^,^15^. These five B populations were founded in February 1980 from a single generation of the “IV” stock studied by Rose and Charlesworth^16^,^17^, which was in turn founded in August 1975 from a sample of 200 wild-caught *Drosophila melanogaster* females obtained by Phillip Ives from his long-studied South Amherst, Massachusetts endemic population^18^. From their first founding, IV and B populations have been cultured using 14-day discrete generations, with mixing of flies across all culture vessels *within* replicate populations, at temperatures of 23–25 degrees Celsius. Effective populations size (*N*_*e*_) are around ~1000 based on calculations from demographic data^13^.

The B populations were pooled and sampled for sequencing at generation 785 from their founding in 1980, in March 2010, though this was after a total of 915 generations of lab domestication since Ives supplied the wild-caught females for founding the laboratory IV stock. From August of 1975 to June of 1981, the IV and B populations were cultured using corn meal based medium in 16 glass milk pint bottles per population, with 12 L:12D light exposure. From June 1981 to March 2010, the B populations sequenced here were cultured in 40 shell vials at densities of 60–80 eggs per vial, yielding 50–75 adults per vial. This equates to around a minimum census size of 2000 each generation. During this period, these B populations were cultured using banana-molasses medium^1^ with 24 L:0D light exposure^14^.

The pooled genomic DNA was obtained by isolating 250 female flies from each of the five population replicates of the B flies, with harvesting ten days after the pupal stage, using the Gentra Puregene Cell Kit (Qiagen, Valencia, CA) according to standard protocol, after maceration of the fly tissues using Dounce Tissue Grinders (Daigger, Vernon Hill, IL). Genomic DNA concentrations and purities of the samples were assayed by DNA spectrophotometer. Size distributions were visualized by low agarose gel electrophoresis with DNA size markers. Genomic DNAs were stored at −20 °C before shipment to Beckman Coulter (Brea, CA) for Illumina paired end sequencing. Reads were 76 bp in length. The fastq files used in our analyses are available through NCBI SRA (BioProject ID: PRJNA350701).

### Mapping of reads

We mapped reads with BWA (version 0.7.8)^19^ against the *D. melanogaster* reference genome (version 5.55) using bwa mem^20^ with default settings. We filtered and sorted the resulting SAM files for reads mapped in proper pairs with a minimum mapping quality of 20 using and converted them to the BAM using the view and sort commands in SAMtools^21^. These files were then converted to mpileup format once again using SAMtools. Using the PoPoolation2^22^ software package, these files were converted to “synchronized” files, which is a format that allele counts for all bases in the reference genome and for all populations being analyzed. Lastly, we used RepeatMasker 4.0.3 ( http://www.repeatmasker.org)^23^ to create a gff file to mask simple sequence repeats and transposable elements of the *D. melanogaster* genome version 5.55.

A table with major and minor allele counts for each SNP in each population was then generated from this synchronized file. SNPs where discarded if coverage in any of the populations was less than 20X or greater than 150X. We also required a minimum minor allele frequency of 2% across all five populations. Based on these parameters, ~1.2 million SNPs were identified across the major chromosome arms. The average coverage at each called SNP was 62X, 65X, 57X, 66X, and 69X in B_1_ through B_5_ respectively.

### Characterizing genetic variation

Local depressions in genetic variation are considered one of the primary means of detecting selective sweeps from population level data^24^. We calculated and plotted heterozygosity across the five major chromosome arms to see if we could find any such evidence for such depressions in our real and simulated data sets. Heterozygosities were calculated over 100 kb non-overlapping windows directly from the major and minor counts in our SNP table. Watterson theta (ϴ) was also calculated using *PoPoolation*^25^, where the details of these calculations can be found. Mpileups were first made for each population using the bam files mentioned above. We then subsampled (without replacement) to a uniform coverage level of 30X across all populations, as these calculations can be sensitive to coverage variation. Estimates of genetic parameters were then calculated over 100 kb non-overlapping windows across the major chromosome arms. For a SNP to be called at a given position, we required a minimum minor allele count of 2. Lastly, sufficient coverage (30X) was required for at least 60% of our 100 kb windows for estimates to be generated.

### F_ST_ estimates

*F*_*ST*_ estimates were obtained using the formula: 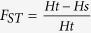 where H_T_ is heterozygosity based on total population allele frequencies, and H_S_ is the average subpopulation heterozygosity in each of the B populations^26^. *F*_*ST*_ estimates were made at every polymorphic site in the data set. This was done to quantify the levels of differentiation between our five B populations, as well as between replicate populations in our simulated scenarios. *F*_*ST*_ estimates were calculated for the B populations along chromosome 3 R at every polymorphic site. This was also done for the simulated data, and once again all polymorphic positions along 3 R not present in the SNP table created from our real data set were discarded.

We also used the formula: 
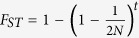
 to generate a predicted *F*_*ST*_ value where *N* is the effective population size of each subpopulation and *t* is the time since divergence from their ancestral population^27^. This model assumes that population diverge randomly over time and that there is no migration. In the event that there is some level of migration between our populations, we used the formula: 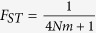 where m is equal to the migration rate and the quantity *Nm* is equal to the number of migrants per generation^27^,^28^. This model assumes no mutation and that the migration rate is small. As with the previous model, it corresponds to a scenario where a single population is split into subpopulations at some point and diverges randomly over time. But in this scenario, migration has placed a limit on how much these subpopulations can diverge and assumes that the populations have reached this limit and are at equilibrium. It is worth noting that this assumption of equilibrium might not be met in our system.

### Simulations

To perform our first set of simulations we used MimicrEE ( https://sourceforge.net/projects/mimicree/)^29^, a forward simulation specifically designed to mimic experimental evolution. It simulates populations of diploid individuals where genomes are provided as haplotypes with two haplotypes constituting a diploid genome. There are no changes in the demography once the initial population file is submitted and a list of selected loci may be provided.

For each selected locus, the selection coefficient (*s*), the dominance coefficient (*h*), and the nucleotide of the nonselected allele are provided (*w*_11_). The fitness of the heterozygous and homozygous individuals is given by: w_11_ = 1, w_12_ = 1 + *hs*, and w_22_ = 1 + *s*^30^. The simulation assumes multiplicative fitness when several selected loci are specified. No *de novo* mutations are considered, as its purpose is to simulate scenarios where adaptation results from selection on standing genetic variation. The simulated populations have non-overlapping generations and all individuals are hermaphrodites (though selfing is excluded). At each generation, matings are performed, where mating success (number of offspring) scales linearly with fitness, until the total number of offspring in the population equals the targeted population size (fecundity selection). Each parent contributes a single gamete to the offspring. Crossing-over events are introduced according to a user-specified recombination rate.

To generate our starting haplotypes, we started with 105 individuals from the Drosophila Genetics Reference Panel (DGRP)^31^. We only used positions along chromosome 3 R and only sites that were polymorphic in the B populations. In total, there were 238,291 polymorphic sites after this filtering. From these 105 haplotypes, we randomly sampled with replacement to 1000 to achieve our desired population size. Recombination rates were specified for 100 kb windows and were obtained from the *D. melanogaster* recombination rate calculator v2.2^32^. As recombination does not occurs in male Drosophila, the empirically estimated female recombination rate was divided by two for the simulations.

We first performed neutral simulations, featuring only drift and recombination, to establish a baseline. We then ran simulations across a variety of evolutionary scenarios that involved different numbers of selected loci. Our goal was to see which, if any, of these scenarios would produce the sorts of patterns we observer in our real data set. For each scenario, we simulated five populations for 800 generations. This was then done 100 times for each scenario. In our selection scenarios, we simulated populations with 5, 10, or 20 randomly distributed beneficial sites. For one set of simulations, the reference nucleotide (A_1_) was defined as the beneficial allele in each case and was also defined as dominant (*h* = 0). For another set, the A_2_ allele was defined as beneficial and dominant (*h* = 1). And for a final set, all selected loci were codominant (*h* = 0.5). For each set, we simulated scenarios with selection coefficients ranging from 0.03 (low) to 0.1 (high) (Table 1). As we increased the number of selected sites, we reduced the selection coefficients to generate scenarios with either few sites of large effect or many sites of small effect. Lastly, we simulated scenarios featuring sites with overdominance to see if extensive balancing selection could be behind the patterns we observe in our data set. In these scenarios, we simulated populations with 20 or 30 randomly distributed sites with overdominance. And once again, we simulated scenarios with a range of selection coefficients (Table 1).

From each set of 5 simulated populations under each scenario, we calculated average heterozygosity and average F_ST_ across all polymorphic sites to compare to values observed in the B populations. We also looked at heterozygosity and F_ST_ over 50 kb windows, and calculated the variance between windows as a means of comparing spatial variation in heterozygosity and F_ST_ to what we observe in the B populations.

### Simulations with migration

Given how long our populations have been maintained in the lab, it is easy to imagine that there may have been some instances of migration due to accidental cross-contamination. Thus, in addition to the selection scenarios mentioned above, we performed simulations featuring migration. These simulations also feature optimizing selection, as opposed to our other simulations featuring directional and over dominant selection. We once again simulated an evolve-and-resequence experiment for a 63 cM long *D. melanogaster* chromosome 3 R. An R program was created to simulate an initial population of *F* founder chromosomes expanded and used to found five populations that were then evolved for *G* generations at a population size of *N* gametes per population with *M* migrant gametes in the meta-population per generation. The simulation was accomplished by tracking founder segments and recombination breakpoints over time. So, the *N* gametes used to found a subpopulation initially consist of a random sample of size *N* drawn from the numbers 1 through *F* with replacement. Then each generation to create *N* new gametes (*n* = 1 … *N*) we draw two gametes with replacement from the previous generation and create a recombination breakpoint at position *r* = unif (0, 1), if *r* < 0.63 (to simulate a chromosome of 63 cM) and *n* modulus 2 equal zero (since recombination only takes place in females). Recombinant chromosomes are represented as a pair of vectors: a founder state vector, and a recombination breakpoint vector. So, for example, the *n*^*th*^ gamete in a population might be ***S***_***n***_ = {3, 17, 31, 3} and ***B***_***n***_ = {0.20, 0.25, 0.60}, indicating that gamete has material from founder #3 from 0 to 20 cM, founder 17 from 20 to 25 cM, etc. This sampling scheme models drift and recombination in a Wright-Fisher population.

Next we added selection to the simulation. We simulated *Q* evenly spaced quantitative trait loci (QTL) on the chromosome, with a vector of effect sizes and starting allele frequencies (***E*** and ***F***), with QTL states randomly assigned via binomial sampling given *F*_*q*_ for each locus. The resulting quantitative trait has a heritability due to the QTL on 3 R of 12%, and an additional polygenic heritability of 38% due to the other chromosomes, and a total phenotypic variance of one. We then model a gamete’s phenotype as the sum of its effect sizes plus a random Gaussian deviate representing a polygenic component, plus a second random Gaussian deviate representing an environmental deviation, plus the current polygenic mean of the population. The fitness of each gamete is a standard Gaussian fitness function proportional to *w* = (pheno − NewOptimum)^2/(2*VarianceFitness), normalized to total fitness. Under this selection scheme each generation gametes are resampled proportional to *w*, resulting in allele frequency changes at the underlying QTL, and resulting changes in the mean phenotype due to those QTL. Furthermore, each generation the polygenic mean (the mean phenotype due to chromosomes other than 3 R) changes according to the Breeder’s equation^33^. That is we partition the trait variance into the variance due to tracked loci (each having V_a,i_ = 2p_i_q_i_a_i_^2^), a polygenic component with Gaussian variance V_a;poly_, and environmental variation (V_e_). We held V_e_/V_t_ constant, but allowed the ratio of V_a_ to V_a;poly_ to vary. Each generation an individual’s phenotypic value is the sum of allelic effects due to tracked loci, a Gaussian deviation due to the polygenic component, and a Gaussian environmental deviate. Those phenotypic values determines a vector of length N consisting of each individual’s average fitness based on the Gaussian fitness function, with N individual’s chosen with replacement from that vector to create the next generation, with the probability of being chosen proportional to average fitness. Between generations, the mean of the population then shifts due to changes in allele frequencies at *both* the tracked loci *and* untracked loci, as predicted by the Breeder’s equation: h^2^_poly_ * S. In this context, h^2^_poly_ is V_a;poly_/(V_a;poly_ + V_e_) and S is the observed selective differential (i.e., the mean phenotype of individuals chosen to reproduce minus the mean of the population). By modeling adaptation in this fashion, the population approaches the new optimum due to genetic changes at both tracked and untracked loci. This model thus accommodates adaptation at both the explicitly modeled chromosome arm and the remainder of the genome.

Under our model, the overall rate at which the mean phenotype changes in the population is controlled by the distance to the new phenotypic optimum and the variance in fitness, which are set to 15 and 12, respectively. Parameters scaling results in *V*_*e*_ = 0.5 and *V*_*p*_ = 1.0 at generation zero, with a new optimum that is 15 phenotype standard deviations away from the population mean, a shift in optimum that was chosen to match experimental evolution experiments in Drosophila (vid. Teotonio and Rose^34^). The simulation was set up so that a population can reach a new optimum phenotype before all underlying QTL are fixed.

Each generation we simulated migration by randomly taking M pairs of gametes in the 5*N set being tracked and replacing the first member of the pair with the second. This corresponds to a one-way island model of migration. This means that if the entire population is 5*N gametes (where 5 is the number of populations), each generation k gametes are chosen and they essentially overwrite K other gametes. That is to say, “one-way” means gametes are not exchanged between populations and “island” means migration is equally likely between any two demes in any direction. We iterated this entire process for *G* generations to obtain a final set of 5*N gametes. Given the relatively short time scale of the experimental evolution experiment, and the relatively modest number of gametes, this simulation is fairly efficient in R on a desktop computer. At the end of the simulation we took a set of 100 (F) 3 R chromosomes from the DGRP^31^, and for each polymorphic position in the real DGRP data we calculated an allele frequency at that site based our simulated populations. This was done because, as with our previous simulations, our starting haplotypes were based on lines from the DGRP. Performing this calculation required a function that maps physical position in bp to cM, and then simply iterates over the founder states at each of the 5*N gametes for each SNP. Despite the fact that poolseq estimates allele frequency in the population based on a finite sample of gametes, with the accuracy of that estimate a function of coverage depth and number of gametes sampled for the Illumina library, we used the exact allele frequency estimates calculated using the method described above in our downstream calculations. Since libraries are made using a large number of individuals (>200) and the per site coverage approaches 60X, this simplification is likely acceptable.

Using this framework, we simulated a number of evolutionary scenarios that involved varying the following: migration rates (M), number of selected QTL, effect sizes of selected QTL, and starting frequencies of selected QTL. Scenarios were simulated using groups of five populations to mimic our observed fly populations. Each scenario was simulated 300 times. All simulations ran for 800 generations (G) and all simulated populations consisted of 2000 gametes (N). We simulated scenarios with 0, 1 or 5 migration events per generation (M). We ran simulations with 0 (i.e., a control with only random genetic drift), 3, 10 and 20 selected QTL. Selected QTL were evenly distributed across the chromosome arm. We also looked at the effects of the starting frequency of selected QTL (F) by running simulations where all selected alleles started at either 0.05 or 0.5. As we increased the number of QTL, we reduced their effect sizes (E) so that the sum of squared effect sizes was held constant. This was done to prevent changes in the heritability of the character. For simulations with 3 QTL, effect sizes were 1, 2, and 2. For simulations with 10 QTL, they were 1, 1, 1, 1, 1, 1, 1, 1, 0.71, and 0.71. And lastly, they were 0.71, 0.71, 0.71, 0.71, 0.71, 0.71, 0.71, 0.71, 0.71, 0.71, 0.71, 0.71, 0.71, 0.71, 0.71, 0.71, 0.5, 0.5, 0.5, and 0.5 for simulations with 20 QTL. [See Table 2 for all simulated scenarios].

Once again, we calculated average heterozygosity and average F_ST_ across all polymorphic sites to compare with the values observed in the B populations. We then again looked at heterozygosity and F_ST_ over 100 kb windows and calculated the variance between windows as a means of comparing spatial variation in heterozygosity and F_ST_ to what we observe in the B populations.

### Selection Detection

To test for footprints of selection across the genome, we relied on a hidden Markov Model developed with the intention to detect sweeps in pooled sequence data, developed by Boitard *et al*.^35^, implemented in the Pool-HMM software package^36^. Their method involves estimating the allele frequency spectrum (AFS) across genomic regions and detecting distortions relative to the background AFS, which are expected to occur in regions subject to selection. Though it is worth noting that while this method was developed primarily to detect selective sweeps, it could be instead identifying signatures of any process that produces similar perturbations of the AFS.

Mpileups for individual populations were used as test inputs, since Pool-HMM can only process data from one population at a time. Scans were performed along each of the major chromosome arms using the following parameters: -n 500 (number of chromosomes in each pool), -pred (predicts the hidden state, “Neutral” (far away from sweep site), “Selected” (close to sweep site), or “Intermediate” (between Neutral and Selected) of each SNP), -C 150 (maximum coverage allowed for sites used in this analysis), -r 5 (where 1/r is the proportion of sites that are used to estimate the background AFS), -theta ϴ (average ϴ for each population was approximately 0.003 based on estimates from PoPoolation, and increasing or decreasing window size did not affect this result), and −k 10^−10^. The −k parameter is the per site transition probability *q* between neutral and selected states, which is an important tuning parameter for the hidden Markov model underlying this test. As *q* increases, less evidence is required for a transition to selection and more sweep candidates should be detected. We also ran tests under more (*q* = 10^−11^) and less stringent (*q* = 10^−9^) conditions, which only led to slight differences in the number of footprints detected (Table 3). A confidence index was calculated for each selective sweep window detected using this method as −log_10_(1 − *p*), where *p* is the maximum of the posterior probability of hidden state “Selection” within the window.

We applied this Pool-HMM test to results from our neutral simulations using the settings listed above, as a means of evaluating our false positive rate. For such tests of simulated data, we used 100 kb regions extracted from different runs of our neutral simulations. Essentially, we converted output from the simulations to mpileup files. Sequence and coverage variation were introduced based on what was found in the actual 3 R sequences from the B populations. In addition to the 100 kb regions just mentioned, we ran tests for selection on results for the entirety of 3 R from 15 simulated populations each taken from a different evolutionary scenario. We did this for populations taken from each of following scenarios: neutral evolution with migration rates M = 0, 5 and 20; 3 selected QTL’s with low starting frequencies with M = 0, 5 and 20; 3 selected QTL’s with high starting frequencies with M = 0, 5 and 20; 10 selected QTL’s with low starting frequencies with M = 0, 5 and 20; 10 selected QTL’s with high starting frequencies with M = 0, 5 and 20.

## Results

### Genetic Variation

**Figure 1:**
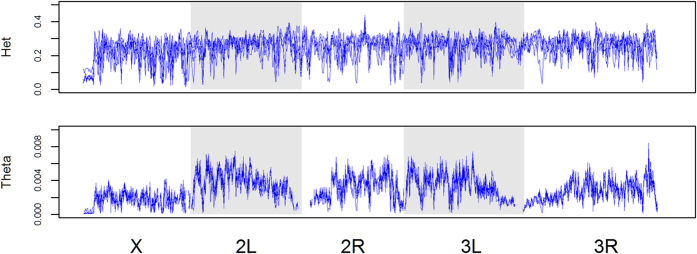
Heterozygosity and Watterson theta (ϴ) plotted across 100 kb non-overlapping windows across all major chromosome arms for the 5 B populations. All replicates are shown.

Plotting measures of genetic variation, heterozygosity and Watterson theta (ϴ), across 100 kb non-overlapping windows reveals depressions in genetic variation across all major chromosomes arms in the B populations (Fig. 1, see Supplementary Fig. S1. for mean heterozygosity and ϴ across all 5 populations). This pattern is robust to both increased (150 kb) and decreased (30 and 50 kb) window size (Supplementary Figs S2–S4). Many depressions in heterozygosity are consistent across the 5 replicate populations, which may be indicative of selection on standing variation. In general, there is a great deal of similarity in patterns of heterozygosity across replicates (Supplementary Fig. S5 shows pair-wise comparisons between all replicates). As in Burke *et al*.^1^, we find no regions where genetic variation has been completely expunged in an unambiguous manner. However, there are regions that show very low levels of heterozygosity (0.2) and theta (ϴ < 0.001) consistently across replicates (Supplementary Tables S1 and S2). In addition, the vast majority of these regions are located in chromosome X. Such regions may arise from incomplete selective sweeps or balanced selective equilibria that are close to fixation boundaries. Nonetheless, we have not found cases that conform to the pattern of heterozygosity expected with hard selective sweeps proceeding all the way to fixation^7^, despite almost 1,000 generations of sustained selection.

We used average heterozygosity as our primary measure of variation when comparing our quantitative results from the actual data obtained from the B populations to the corresponding results obtained from the different evolutionary scenarios we simulated. Average heterozygosity in the *starting* base population used in our simulations (see Materials and Methods) was 0.32. Average heterozygosity across 3 R was lower in the five B-population replicate data at 0.28, 0.29, 0.28, 0.27, and 0.27, respectively. We find lower levels of heterozygosity after 800 generations in all of our evolutionary scenarios (Table 1 and Table 2). Spatial variance in heterozygosity along the chromosome arm, based on calculations from 50 kb windows, were as follows for the five B replicates: 0.21, 0.24, 0.24, 0.37, and 0.37.

In our simulations performed using MimicrEE, we found that the addition of selection does result in greater losses in heterozygosity than genetic drift alone, as expected (Table 1). In our scenarios where the A_1_ allele is dominant and beneficial at each selected site, we found that increasing the number of selected sites produced greater reductions in heterozygosity (Table 1). This was also true when the A_2_ allele was dominant or if we used a selection model featuring codominance. In terms of spatial variance in heterozygosity, we find the highest levels in scenarios featuring strong selection at 3 (0.055 to 0.060) sites or weaker selection at 20 sites (Table 1). The lowest levels of spatial variance, which were comparable to the higher end of what was observed in the B populations, were found in scenarios with selection at 10 sites (0.0035 to 0.0040).

In our scenarios with overdominance, we found that increasing the number of sites under selection and increasing selection coefficients both produce greater reductions in heterozygosity near locations undergoing selection. Heterozygosity was maintained at the selected sites themselves and selected alleles approach predicted equilibrium frequencies based on our settings, but heterozygosity was nonetheless reduced in surrounding regions. We also found spatial variance in heterozygosity to be higher than what we typically observed in the B populations, with the exception of scenarios featuring 30 sites with small selection coefficients (0.34) (Table 1).

In our simulations with migration, we once again found that the addition of selection results in greater reductions in heterozygosity overall (Table 2). Increasing the number of selected QTLs again produced a greater decrease in heterozygosity. Increasing migrations rates resulted in more variation being maintained, as did increasing the starting frequencies of selected QTLs; however, the simulated levels of genetic variation never achieved the levels seen in the actual B populations. In terms of genome-wide variance in heterozygosity, allowing migration and increasing the starting frequencies of selected QTLs produced results closer to those observed in the B populations (Table 2).

### F_ST_

Mean *F*_*ST*_ across the 5 B populations was 0.08 across all chromosome arms, including 3 R individually. This value is far lower than what we would predict assuming no migration using the formula 
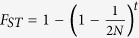
, which predicts *F*_*ST*_should be around 0.33 assuming N = 1,000 and t ~ 800 generations. Substituting our observed *F*_*ST*_ into this equation and instead solving for N suggests that in order to produce an *F*_*ST*_ estimate of 0.08, assuming no migration and random divergence, we would have to have an effective population size of around 4,700. To assesses how much migration would be required to produce this result, assuming random divergence and populations at equilibrium, we used the formula 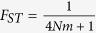. Solving for *Nm*, the number of migrants per generations, suggests our observed *F*_*ST*_ could be produced if there were 2.88 successful migrants per generation, each and every generation, if the assumptions of this model are met.

Our observed *F*_*ST*_was also far lower than anything produced in the different evolutionary scenarios we simulated (Tables 1 and 2). In our simulations performed using MimicrEE, we found that scenarios where A_1_ allele was dominant and beneficial at each selected site all produced greater *F*_*ST*_ estimates than were produced by drift alone. Scenarios with overdominance and low selection coefficients produced modest reductions in mean *F*_*ST*_, but never to the level observed in the B populations. This effect was lost when selection coefficients were increased, once again giving mean *F*_*ST*_ estimates greater than those produced by drift alone. All simulations also produced much greater variance in genome-wide *F*_*ST*_, 0.0028 at the lowest, than we observe in the B populations (0.0004), for the single chromosome arm.

In our simulations featuring migration, increasing the number of QTL under selection and/or altering the starting frequencies of favored genotypes both failed to have any appreciable effects on mean *F*_*ST*_ (Table 2). Increasing the migration rates did reduce *F*_*ST*_ as expected. However, even with migration rates as great as 5 gametes per generation, far higher than we consider likely, *F*_*ST*_ estimates failed to approach the values observed in the actual B populations. Increasing migration rates also reduced variance in F_ST_ along the chromosome arm, but once again not to the levels observed in the B populations (Table 2).

### Footprints of Selection

**Figure 2:**
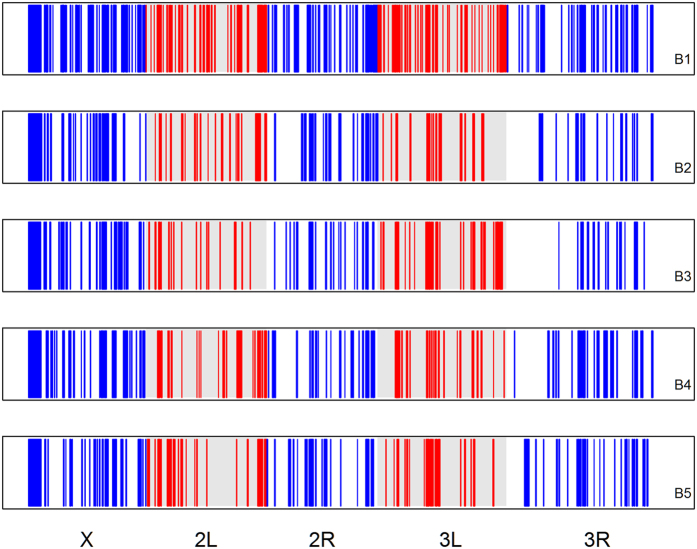
Regions across all major chromosome arms in the 5 B populations showing evidence for selection based on our analysis using Pool-Hmm. Each panel shows results from a different B population replicate. There is no significance to the color coding outside other than differentiating adjacent chromosome arms.

**Figure 3:**
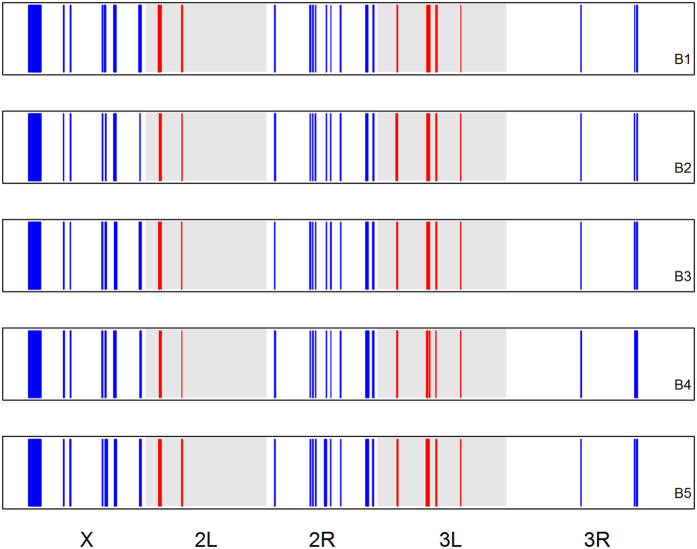
Overlapping regions across all major chromosome arms showing evidence for selection across all 5 B populations based on our analysis using Pool-Hmm. Each panel shows results from a different B population replicate as these regions do not perfectly overlap. There is no significance to the color coding other than differentiating adjacent chromosome arms.

We used the pool-HMM method^36^ to detect selective sweeps or changes in allele frequency due to selection across all major chromosome arms in the B populations. When applying Pool-HMM to our real sequence data, we detected dozens of signatures of selection on each of the major chromosome arms for all of the B populations (Fig. 2 and Table 3). Additionally, nearly twice as many regions were detected in the B_1_ population compared to the other four replicates. Of the hundreds of candidate selected regions detected, there were ~35 regions that overlapped across all five replicates (Fig. 3). However, as many of these regions were in excess of 100 kb, these results do not definitively point to any specific genes as being targets of selection.

We also applied pool-HMM to results from our neutral simulations, both those including and those excluding migration. We applied the test to regions consisting of 100 kb sampled from our neutral simulations using the same settings we applied to the data from the B populations. Using these settings, we found very few instances where selection was detected, suggesting a low false positive rate (Supplementary Table S3). For instance, we only found 2 instances where selection was falsely detected after applying pool-HMM to 300 100 kb regions sampled from our neutral simulations with no migration. This was also true for 300 regions sampled from neutral simulations with M = 1. Lastly, when M = 5 there were zero instances where selection was falsely detected.

We also ran pool-HMM on results from the entirety of 3 R for several of the simulated neutral and selective scenarios we tested. We did this for one simulated population from each of the following scenarios: neutral evolution with M = 0, 5 and 20; 3 selected QTL’s with low starting frequencies with M = 0, 5 and 20; 3 selected QTL’s with high starting frequencies with M = 0, 5 and 20; 10 selected QTL’s with low starting frequencies with M = 0, 5 and 20; 10 selected QTL’s with high starting frequencies with M = 0, 5 and 20. Once again, few regions were identified as being under selection when pool-HMM was applied to results from our neutral simulations relative to what we observe in the B populations. Seven regions in total were detected when we applied it to results from a neutral simulation with M = 0, 2 regions when M = 5, and 0 when M = 20 (Supplementary Fig. S6).

However, we found pool-HMM’s ability to detect selected QTL to be highly dependent on the starting frequency of the selected QTL and on assumed migration rates. When starting frequencies are low (0.05) and there is no migration, there is some correspondence between the regions identified by pool-HMM and the locations of the actual selected QTL with pool-HMM identifying regions overlapping or adjacent to selected QTL (Supplementary Figs S7 and S8). However, when the starting frequencies of selected QTL were high (0.5), this correspondence broke down (Supplementary Figs S9 and S10). For instance, when we applied pool-HMM to results from a simulation with zero migration and 10 selected QTLs starting at low frequency, 11 regions were identified as being under selection by pool-HMM (Supplementary Fig. S8). Five of these regions directly overlapped with the locations of our selected QTLs, and the remaining regions were adjacent to selected QTL. However, when we applied pool-HMM to results from a simulation with zero migration and 10 selected QTLs starting at high frequency, only 3 regions were detected (Supplementary Fig. S10). Of these 3 regions, only one overlapped with the location of a selected QTL.

Migration also had a pronounced effect on pool-HMM’s ability to detected selected QTL. Across all the scenarios we tested, we found that increased migration rates resulted in reductions in the number of regions identified as being under selection (Supplementary Figs S6–S10). As mentioned previously, in scenarios with 10 selected QTL’s starting at low frequencies, 11 regions were detected when M = 0. However, when M = 20 only 4 regions were detected and only one of those overlapped with the location of a selected QTL (Supplementary Fig. S8). Combining high migration rates and high starting frequencies further impaired pool-HMM ability to detect selected QTL. For instance, when M = 5 for simulations with 10 QTL starting at high frequencies, pool-HMM did not identify any regions as being under selection.

In summary, we found that both migration and the starting frequency of selected alleles affect the rate and accuracy at which pool-HMM identifies regions as being under selection. Consequently, the overall correspondence between regions identified by pool-HMM and the location of selected QTL in our simulated results was generally poor. Pool-HMM also detected far fewer regions in our simulated results than in any of the scans of our real data from the B population. This suggests there may be some other evolutionary factor(s) behind the allele frequency distributions in the evolved populations other than those we simulated (selection at modest number of sites, migration, and drift). It is not entirely clear what this factor might be from our results. For instance, this discrepancy could be the result of some demographic factor acting on the B populations or some selective scenario not tested. Or perhaps a combination of the two.

## Discussion

Applying all our measures of genetic variation to the five observed *Drosophila* populations, we found some depressions indicating reduced genetic polymorphism. But there are no regions where it was completely expunged. When comparing these results to the combined DGRP lines we used as base populations for our simulations, we found levels of variation in our populations to be lower on average. While there are clearly other factors at play, this disparity could also be due in large part to the nearly 1000 generations of evolutionary domestication that the experimental B populations have been subjected to, domestication that has featured both reduced effective population sizes as well as long-sustained stable patterns of selection. This hypothesis is supported by the localized reductions in polymorphism found within our populations, reductions which are consistent with adaptation involving allele frequencies moving part-way toward fixation^7^. Given that many of these reductions are consistent across our replicates and genetic variation is never entirely depleted, it also seems reasonable to infer that they result from selection on standing genetic variation.

Our tests for selection using pool-HMM are also suggestive of a widespread response to selection across the genome in our populations. However, it is unclear how many of these regions are indicative of a recent response to selection, or selection in the wild ancestral population sampled by Ives in 1975. We find a number of regions that are consistently implicated across all replicates, which is perhaps indicative of a parallel response to selection. However, further complicating matters, our tests on data from simulated populations suggest demographic factors and the starting frequencies of selected variants can have pronounced effects on pool-HMM’s ability to detect regions under selection. The role of the former in particular warrants further investigation. We found that migration produced large reductions in the number of regions identified as being under selection by pool-HMM across all scenarios we tested. Given the number of regions detected when pool-HMM is applied to the B population sequence data, it seems unlikely that migration between B populations is a major confounding factor. However, it is entirely possible that these results could be due to some other demographic factor or combinations of factors not explored in our simulations.

We find that, after almost 1,000 generations of laboratory cultivation, the five replicate B populations studied here are not generally genetically depauperate. This is somewhat surprising, given the moderate N_e_ estimates of Mueller *et al*.^13^ for these populations: generally a bit less than 1,000. If the only evolutionary processes acting on these populations were selective sweeps, background selection, and genetic drift, then it seems odd that such extensive genetic variation is maintained. The results of our simulation add to this mystery, as we consistently find greater simulated reductions in average heterozygosity than that shown by the B populations, across a range of evolutionary scenarios featuring drift and selection. Note that for more than 900 generations, these populations were maintained under stable conditions with respect to life cycle, illumination, density, and handling vessel. This provided an excellent opportunity for a selective sweep to occur, since the B populations were maintained for a long time in a consistent selection regime, much longer than would be likely to arise in nature.

That being said, our simulations are far from perfect. Given the age of our system, we have no record of the starting genetic make-up of our populations. The populations featured in this study were derived from a single population that had been maintained for 130 generations under laboratory conditions. This population was in turn created from 200 gravid females collected in the wild brought into the lab. In contrast, the base population used in our simulations was created by essentially combining a hundred inbred lines from the DGRP. This difference alone represents a major confounding factor. Additionally, while we feel we are justified in excluding the potential for *de novo* beneficial mutations in our simulations, given the values of our actual *N*_*e*_ and the number of generations under selection, there no doubt remains value in exploring a wider range of evolutionary scenarios. However, addressing these issues satisfactorily would constitute a considerable undertaking, well beyond the scope of this project.

Laboratory selection experiments with *Drosophila* have provided a variety of results and interpretations concerning the underlying mechanisms of adaptation. For instance, both Turner *et al*.^2^ and Zhou *et al*.^37^ report patterns of locally-purged genetic variation in evolved populations consistent with the classic signature of complete selective sweeps. But we do not find any regions where genetic variation is locally purged in the manner associated with a complete selective sweep, as heterozygosity across the genome in our evolved populations never unambiguously achieves zero values in well-defined local regions of the genome. Our findings are more consistent with those of Burke *et al*.^1^, Orozco-terWengel *et al*.^8^ and Tobler *et al*.^38^; the patterns of adaptation that they found were attributed to selection on standing genetic variation, without complete fixation of favored alleles.

This discrepancy may be due to differences in experimental methods. For instance, the study of Turner *et al*.^2^ featured an artificial selection experiment in which flies that met specific body size criteria were selected and allowed to reproduce. Their breeding population sizes were substantially smaller than ours, at 160 females and 160 males. This is in contrast to those experimental evolution studies where there is no direct choice of individuals who will contribute to the next generation, which might have led to very different patterns of evolution. In Zhou *et al*.^37^, the study populations were founded from 27 isogenic lines. Our populations were not created by crossing of inbred lines. The populations studied by Orozco-ter Wengel *et al*.^8^ and Tobler *et al*.^38^ were founded using 113 isofemale lines, and thus should have had far more genetic variation to begin with than the populations studied by Zhou *et al*.^37^, perhaps even more than our founding “Ives” population, which was started with about 200 fertilized females sampled from the wild^14^.

The only published study that is closely comparable to this one is that of Burke *et al*.^1^, also from our laboratory, although that study was somewhat impaired by the use of a single unpooled replicate population alongside two sets of pools of five replicate populations. Nevertheless, it too featured long sustained selection, founding populations that had never been systematically inbred, and five-fold replication of the selected and ancestral treatments. A failure to detect completely depressed heterozygosity in the five-replicate pools of that study could be attributed to differentiation between replicate populations with respect to selective sweeps. However, the single unpooled population (ACO_1_) from the Burke *et al*. study also did not show clear signatures of completed selective sweeps, despite just over 600 generations of sustained selection.

The high degree of similarity in patterns of variation between our five replicate populations is another surprising aspect of our results. We found that our observed level of *F*_*ST*_ was in fact much lower than what would be predicted by classical theory assuming no migration and random divergence between subpopulation. To produce the level of *F*_*ST*_ we observe using this model would require an effective population size nearly five times greater than estimates made from empirical data by Mueller *et al*.^13^, and as such we do not believe this discrepancy can be reasonably explained away by issues with our population size estimates. Our attempts to predict how much migration would be required to produce our observed *F*_*ST,*_ assuming random divergence and populations at equilibrium suggest that ~3 migrants per generation would be sufficient. However, given the nature of our system and its maintenance protocols, migration rates that high *every* generation seem unlikely. It is also worth noting we have no guarantee that the assumption of equilibrium conditions has been met in our populations, which could confound this estimate^39^. And this lack of equilibrium convergence serves as a possible explanation as to why migrations rates of 5 per generation did not produce our observed *F*_*ST*_ in simulated scenarios.

In our simulations, increasing the number of selected sites/QTLs and migration rates both failed to produce comparable F_ST_ estimates to what we find in the five B populations. Increasing migration rates did produce reductions in F_ST,_ and it is likely that a drastic increase in rates of simulated migration per generation would produce something comparable to our observed values. However, once again, migration rates that high *every generation* do not seem likely, given that these populations are maintained independently and all migration is by definition accidental. It would also likely result in too much genetic variation being maintained, relative to the patterns in our genome-wide data, unless population sizes were also reduced. Our results could possibly be explained by parallel selection at a large number of loci, but more work would be required to test this hypothesis. And our initial findings suggest even this explanation may not be adequate.

Regarding the relative importance of selective sweeps, our results are not conclusive. However, the high levels of genetic variation maintained in these populations in the face of relatively small population sizes, which foster genetic drift and background selection^13^, together with the long-sustained selection which should foster reduced genetic variation due to selective sweeps, seem difficult to reconcile with the idea of adaptation primarily driven by hard selective sweeps. If one’s imagination extends so far as to suppose that such selective sweeps arise for a few alleles which are consistently favored by natural selection for far more than 1,000 generations, say for 100,000 generations, then our experiment does not test for the existence of such alleles. We doubt that any laboratory experiment with outbred metazoa will accomplish such a test in this century^40^.

## Additional Information

**How to cite this article**: Phillips, M. A. *et al*. Genome-wide analysis of long-term evolutionary domestication in *Drosophila melanogaster. Sci. Rep.* **6**, 39281; doi: 10.1038/srep39281 (2016).

**Publisher's note:** Springer Nature remains neutral with regard to jurisdictional claims in published maps and institutional affiliations.

## Supplementary Material

Supplementary Information

## Figures and Tables

**Table 1:** 95% confidence intervals for average F_ST_ and average heterozygosity for simulations with unconditionally beneficial alleles and overdominance.

Heterozygous Effect	Number of Sites	Selection Coefficient (s)	Mean Het	Variance Het	Mean *F*_*ST*_	Variance F_ST_
B populations	NA	NA	0.28	0.0024	0.08	0.0004
	NA	NA	0.29	0.0021		
	NA	NA	0.28	0.0024		
	NA	NA	0.27	0.0037		
	NA	NA	0.27	0.0037		
Neutral	NA	NA	0.22	0.0031 ± 7.0 × 10^−5^	0.26	0.0022 ± 8.5 × 10^−5^
Overdominance	20	0.03	0.20	0.0039 ± 5.3 × 10^−5^	0.24	0.0039 ± 0.0002
		0.065	0.19	0.0041 ± 5.0 × 10^−5^	0.24	0.0051 ± 0.0002
		0.1	0.18	0.0042 ± 4.4 × 10^−5^	0.26	0.0066 ± 0.0003
		0.03 < s < 0.1	0.21	0.0032 ± 5.4 × 10^−5^	0.24	0.0028 ± 9.0 × 10^−5^
	30	0.03	0.22	0.0034 ± 8.0 × 10^−5^	0.25	0.0027 ± 0.0001
		0.065	0.20	0.0041 ± 9.8 × 10^−5^	0.27	0.0038 ± 0.0002
		0.1	0.15	0.0060 ± 0.0001	0.42	0.0108 ± 0.0005
		0.03 < s <0 0.1	0.19	0.0040 ± 6.5 × 10^−5^	0.27	0.0045 ± 0.0002
A1 Dominant	5	0.065	0.20	0.0060 ± 0.0002	0.29	0.0055 ± 0.0005
	10	0.0325	0.21	0.0035 ± 7.6 × 10^−5^	0.28	0.0033 ± 0.0001
	20	0.01625	0.19	0.0058 ± 0.0002	0.32	0.0055 ± 0.0002
A2 Dominant	5	0.065	0.20	0.0055 ± 0.0002	0.28	0.0042 ± 0.0002
	10	0.0325	0.20	0.0037 ± 6.1 × 10^−5^	0.27	0.0033 ± 0.0001
	20	0.01625	0.19	0.0050 ± 0.0001	0.30	0.0045 ± 0.0002
Codominant	5	0.065	0.20	0.0060 ± 0.0002	0.29	0.0051 ± 0.0003
	10	0.0325	0.20	0.0040 ± 6.4 × 10^−5^	0.29	0.0041 ± 0.0002
	20	0.01625	0.18	0.0061 ± 0.0002	0.33	0.0060 ± 0.0003

Confidence intervals for each scenario are based on the distribution of these values taken from 100 simulation runs where each run consists of 5 simulated populations.

**Table 2:** Average genome wide F_ST_ and average heterozygosity for B populations and simulations with selection and migration.

Populations/Selection Scenario	Migration Rate	Mean Het	Variance Het	Mean F_ST_	Variance F_ST_
B populations	NA	0.28	0.0024	0.08	0.0004
	NA	0.29	0.0021		
	NA	0.28	0.0024		
	NA	0.27	0.0037		
	NA	0.27	0.0037		
Neutral	M = 0	0.22	0.0025 ± 2.5 × 10^−5^	0.24	0.0021 ± 6.5 × 10^−5^
	M = 1	0.22	0.0024 ± 2.6 × 10^−5^	0.22	0.0020 ± 6.2 × 10^−5^
	M = 5	0.24	0.0020 ± 2.2 × 10^−5^	0.17	0.0012 ± 3.7 × 10^−5^
3 QTLs with 0.05 starting freq.	M = 0	0.19	0.0037 ± 5.3 × 10^−5^	0.24	0.0027 ± 0.0001
	M = 1	0.20	0.0034 ± 4.8 × 10^−5^	0.22	0.0023 ± 8.6 × 10^−5^
	M = 5	0.22	0.0029 ± 4.6 × 10^−5^	0.16	0.0015 ± 6.6 × 10^−5^
3 QTLs with 0.5 starting freq.	M = 0	0.21	0.0025 ± 2.8 × 10^−5^	0.24	0.0023 ± 6.6 × 10^−5^
	M = 1	0.22	0.0024 ± 2.5 × 10^−5^	0.22	0.0021 ± 7.2 × 10^−5^
	M = 5	0.24	0.0020 ± 2.2 × 10^−5^	0.16	0.0013 ± 4.3 × 10^−5^
10 QTLs with 0.05 starting freq.	M = 0	0.16	0.0048 ± 5.2 × 10^−5^	0.26	0.0033 ± 0.0002
	M = 1	0.17	0.0046 ± 6.1 × 10^−5^	0.24	0.0030 ± 0.0002
	M = 5	0.19	0.0039 ± 6.0 × 10^−5^	0.17	0.0018 ± 0.0001
10 QTLs with 0.5 starting freq.	M = 0	0.21	0.0026 ± 3.1 × 10^−5^	0.24	0.0025 ± 9.6 × 10^−5^
	M = 1	0.21	0.0025 ± 2.7 × 10^−5^	0.22	0.0022 ± 9.3 × 10^−5^
	M = 5	0.23	0.0021 ± 2.2 × 10^−5^	0.17	0.0014 ± 5.6 × 10^−5^
20 QTLs with 0.05 starting freq.	M = 0	0.16	0.0050 ± 5.5 × 10^−5^	0.27	0.0044 ± 0.0002
	M = 1	0.17	0.0048 ± 5.4 × 10^−5^	0.24	0.0040 ± 0.0002
	M = 5	0.19	0.0043 ± 6.2 × 10^−5^	0.18	0.0022 ± 0.0001
20 QTLs with 0.5 starting freq.	M = 0	0.21	0.0027 ± 3.4 × 10^−5^	0.24	0.0026 ± 0.0001
	M = 1	0.21	0.0026 ± 2.7 × 10^−5^	0.22	0.0022 ± 7.9 × 10^−5^
	M = 5	0.23	0.0021 ± 2.3 × 10^−5^	0.17	0.0015 ± 5.9 × 10^−5^

For the B populations, variance in heterozygosity and F_ST_ over 50 kb windows is shown. For each simulated scenarios, 95% confidence intervals for variance in heterozygosity and F_ST_ over 50 kb windows calculated from replicate simulation are shown.

**Table 3:** Number of regions where selection was detected using Pool-HMM method with different per site transition probabilities (*q*).

*q* =	B1			B2			B3			B4			B5		
	10^−9^	10^−10^	10^−11^	10^−9^	10^−10^	10^−11^	10^−9^	10^−10^	10^−11^	q10^−9^	10^−10^	10^−11^	10^−9^	10^−10^	10^−11^
2L	68	61	53	41	33	29	30	26	22	40	34	29	39	35	31
2R	70	62	58	44	42	37	40	36	31	38	33	26	36	32	29
3L	79	62	54	32	31	29	52	43	38	42	38	34	32	31	29
3R	75	67	64	37	32	25	27	24	22	51	48	44	49	42	37
X	70	61	53	50	45	43	49	41	35	52	45	43	39	35	32
Total	362	313	282	204	183	163	198	170	148	223	198	176	195	175	158
