# Supplementary material for: Genome-wide analysis of long-term evolutionary domestication in Drosophila melanogaster
Source: Sci Rep. 2016 Dec 22;6:39281. doi: 10.1038/srep39281 (PMC5177908; doi:10.1038/srep39281)
Supplement: Supplementary Information [file srep39281-s1.pdf]

Title: Genome-wide analysis of long-term evolutionary domestication in *Drosophila melanogaster*

Authors*:* Mark A. Phillips, Anthony D. Long, Zachary S. Greenspan, Lee F. Greer, Molly K. Burke, Bryant Villeponteau, Kennedy C. Matsagas, Cristina L. Rizza, Laurence D. Mueller, and Michael R. Rose.

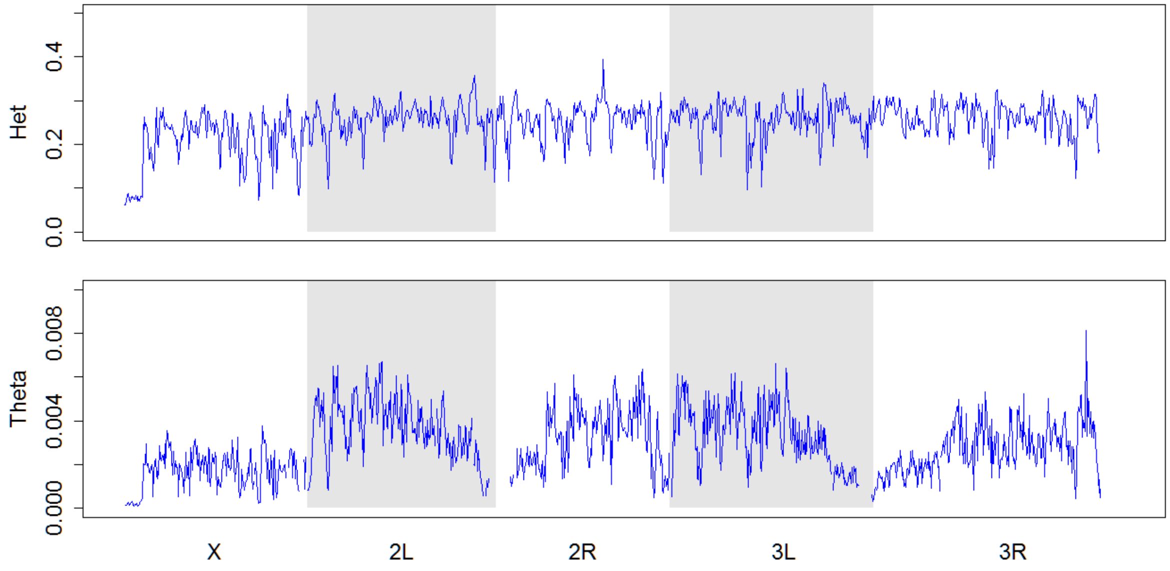

Supplementary Figure S1. Mean heterozygosity and Watterson theta (Θ) plotted across 100kb non-overlapping windows across all major chromosome arms for the 5 B populations.

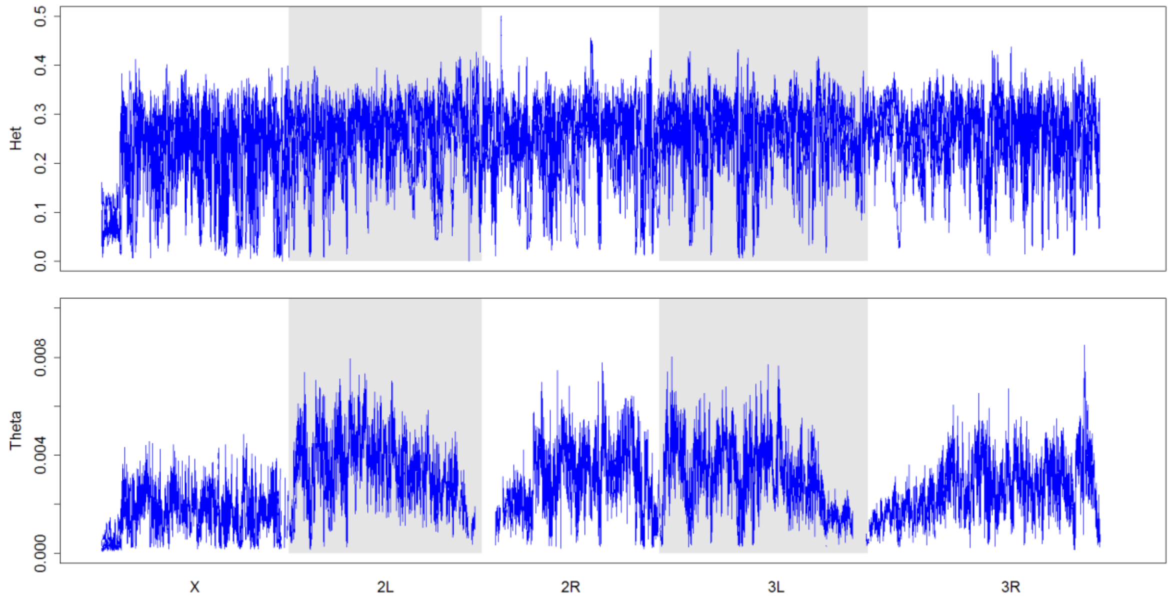

Supplementary Figure S2. Heterozygosity and Watterson theta (ϴ) plotted across 30kb non-overlapping windows across all major chromosome arms for the 5 B populations.

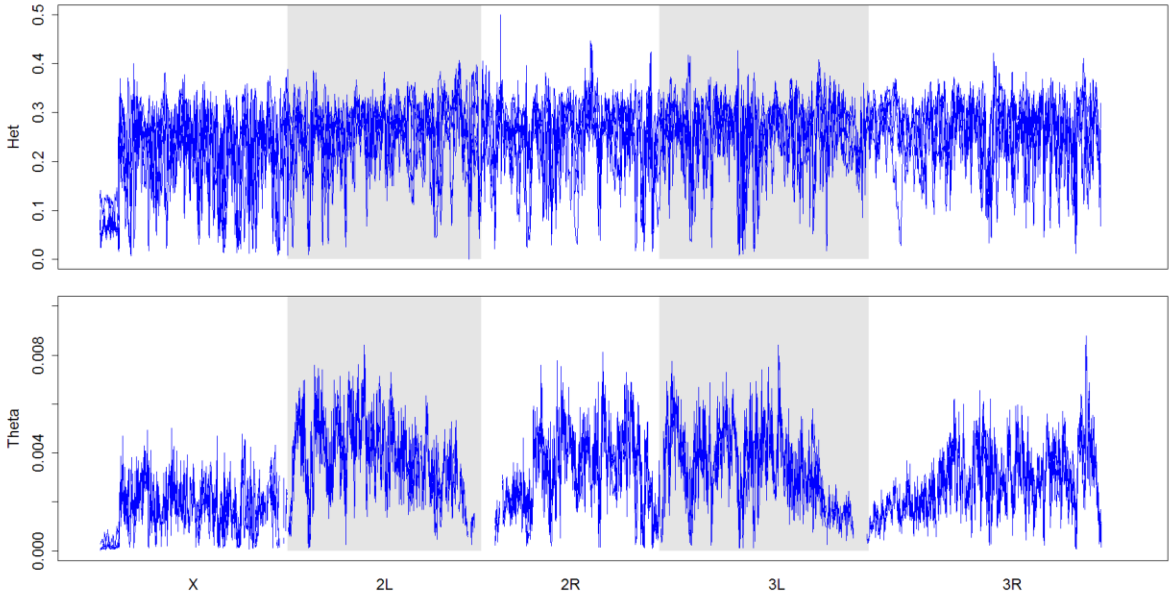

Supplementary Figure S3. Heterozygosity and Watterson theta (ϴ) plotted across 50kb non-overlapping windows across all major chromosome arms for the 5 B populations.

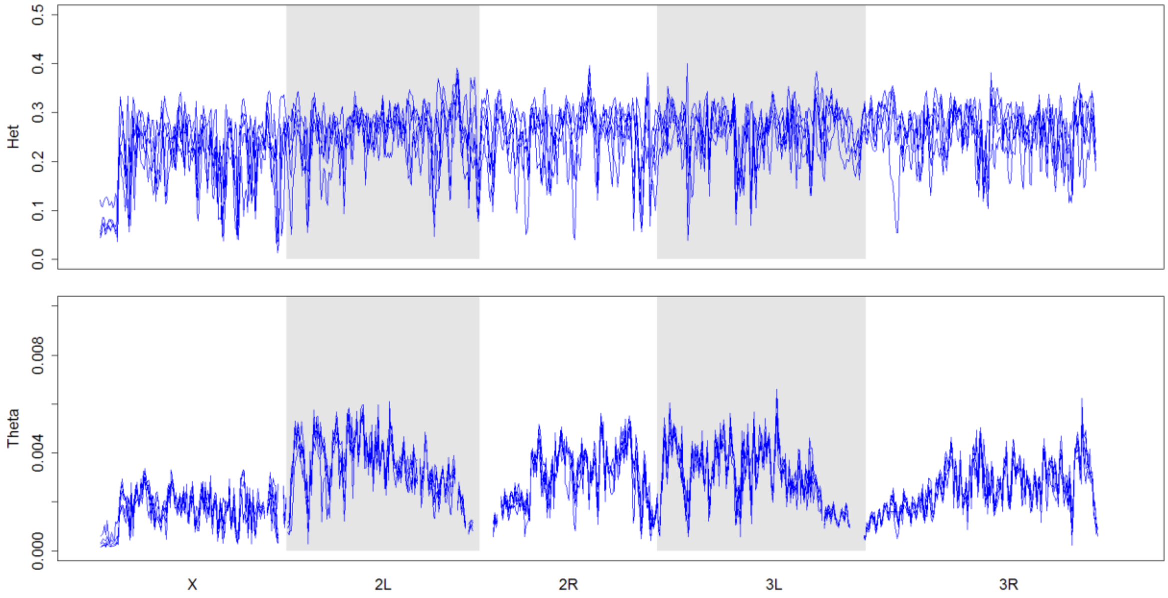

Supplementary Figure S4. Heterozygosity and Watterson theta (Θ) plotted across 150kb non-overlapping windows across all major chromosome arms for the 5 B populations.

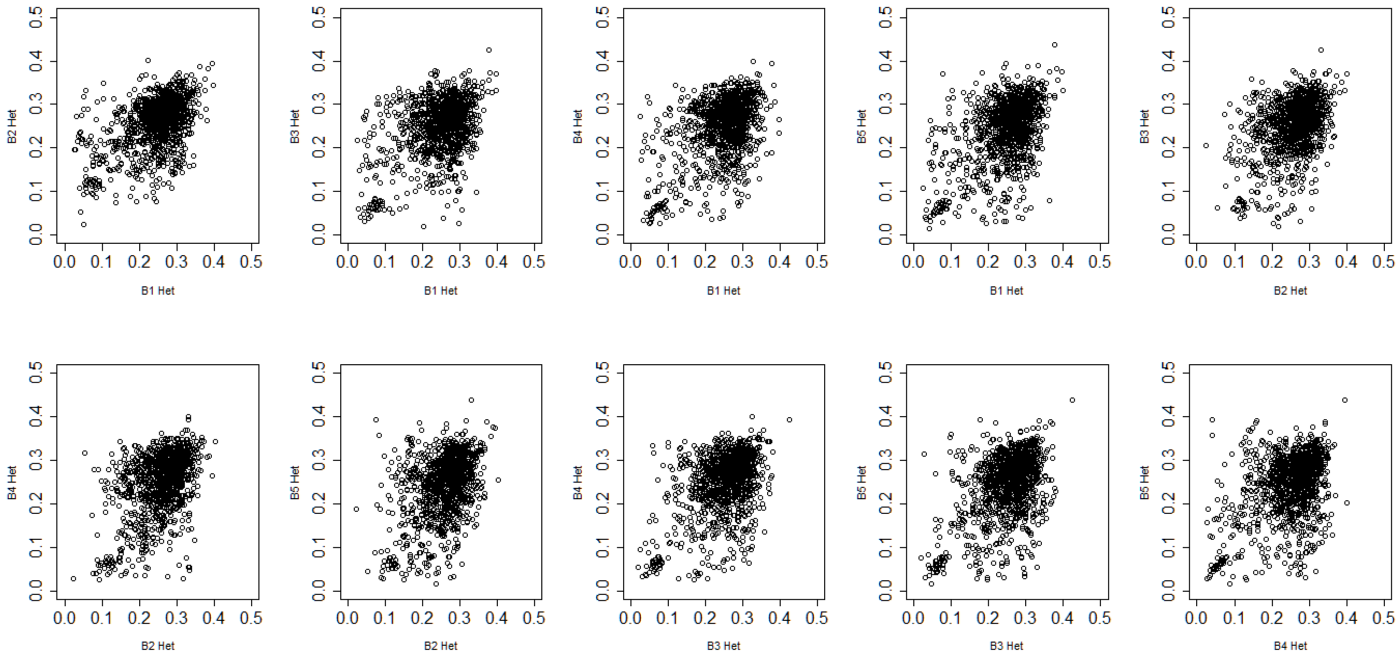

Supplementary Figure S5. Pair-wise comparisons of heterozygosity (calculated over 100kb windows) for all 5 B populations.

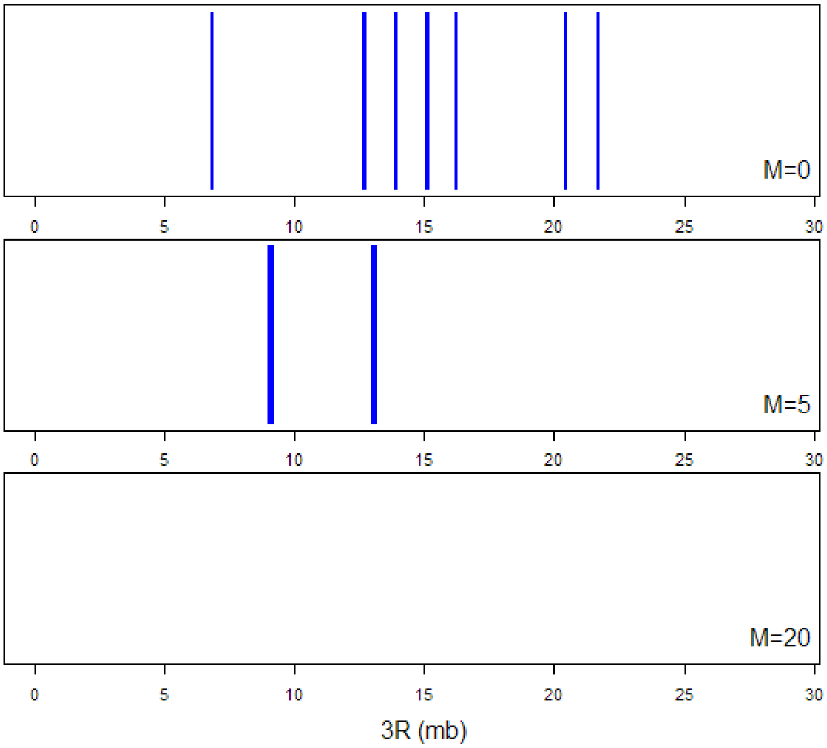

Supplementary Figure S6. Pool-HMM results for chromosome arm 3R from simulations featuring neutrally evolving populations. Regions where selection was detected by the Pool-HMM are shown in blue. Results are shown for a population with no migration (top), a population from a group of 5 where there were 5 migration events per generation, and a population from a group of 5 where there were 20 migration events per generation.

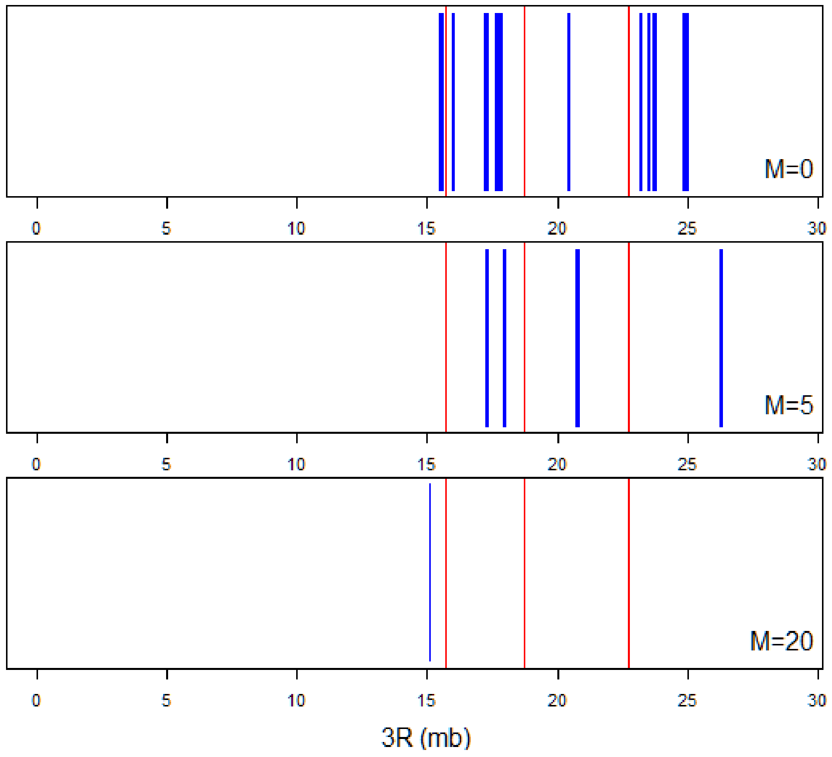

Supplementary Figure S7. Pool-HMM results for chromosome arm 3R from simulations with 3 selected QTL starting at low frequencies (0.05). The locations of the selected QTLs are indicated by red lines, and regions where selection is detected by Pool-HMM are in blue. Results are shown for a population with no migration (top), a population from a group of 5 where there were 5 migration events per generation (middle), and a population from a group of 5 where there were 20 migration events per generation (bottom).

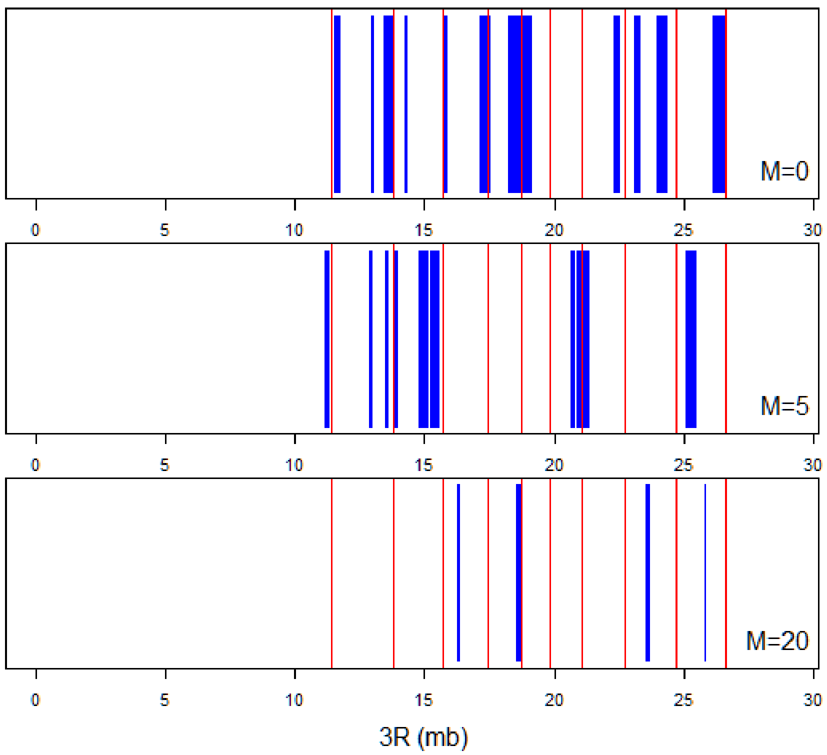

Supplementary Figure S8. Pool-HMM results for simulations with 10 selected QTL starting at low frequencies (0.05). The locations of the selected QTLs are indicated by red lines, and regions where selection is detected by Pool-HMM are in blue. Results are shown for a population with no migration (top), a population from a group of 5 where there were 5 migration events per generation (middle), and a population from a group of 5 where there were 20 migration events per generation (bottom).

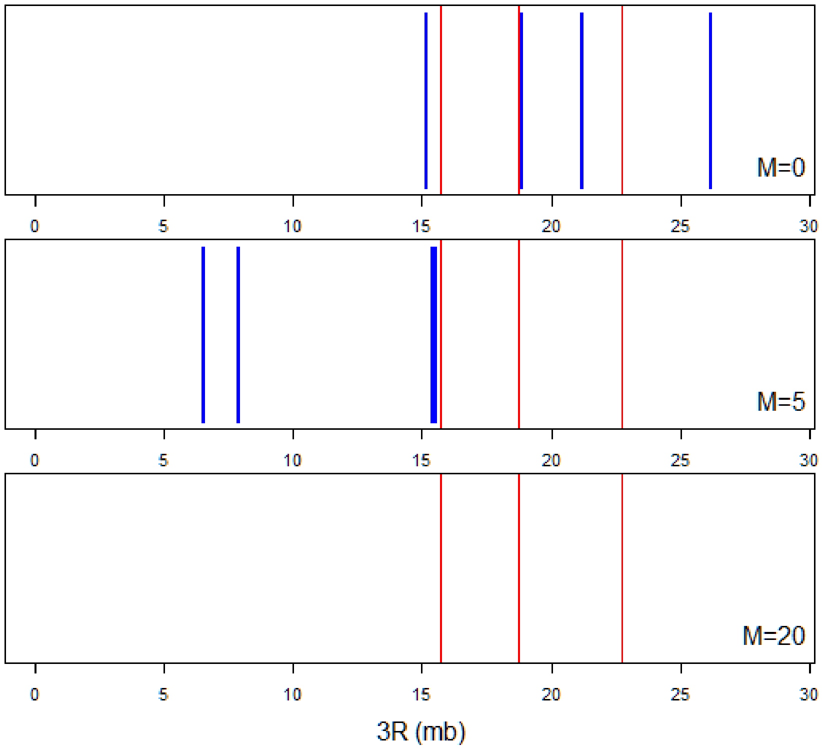

Supplementary Figure S9. Pool-HMM results chromosome arm 3R from simulations with 3 selected QTL starting at high frequencies (0.5). The locations of the selected QTLs are indicated by red lines, and regions where selection is detected by Pool-HMM are in blue. Results are shown for a population with no migration (top), a population from a group of 5 where there were 5 migration events per generation (middle), and a population from a group of 5 where there were 20 migration events per generation (bottom).

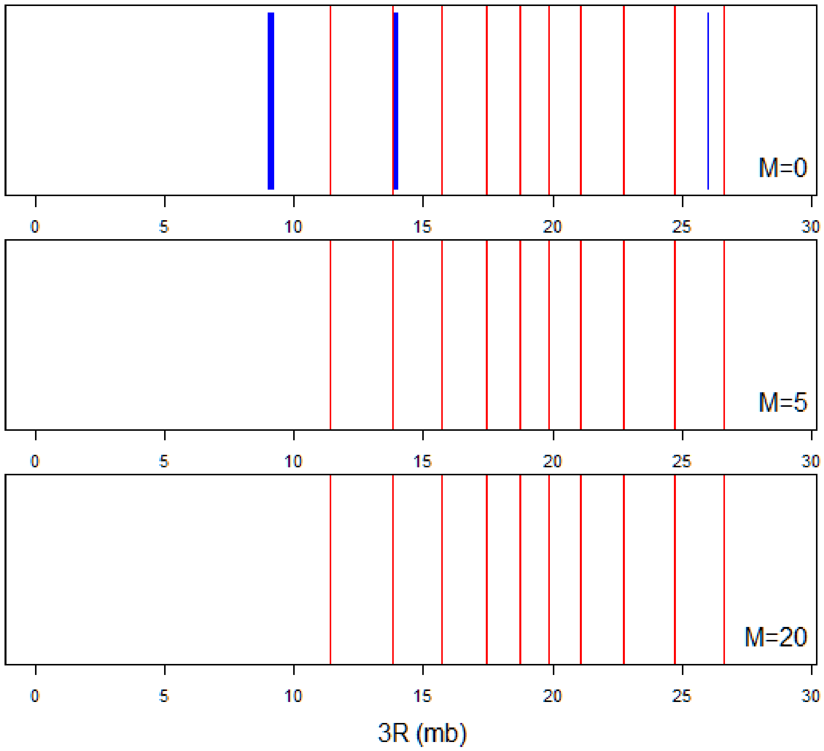

Supplementary Figure S10. Pool-HMM results for simulations with 10 selected QTL starting at high frequencies (0.5). The locations of the selected QTLs are indicated by red lines, and regions where selection is detected by Pool-HMM are in blue. Results are shown for a population with no migration (top), a population from a group of 5 where there were 5 migration events per generation (middle), and a population from a group of 5 where there were 20 migration events per generation (bottom).

Supplementary Table S1. 100 kb regions that consistently have values of ϴ less than 0.001 across all 5 B populations.

Chromosome	Start of region
**2L**	50000
**2L**	21350000
**2L**	21750000
**2R**	19150000
**3L**	150000
**3R**	50000
**3R**	150000
**3R**	250000
**3R**	550000
**3R**	1450000
**3R**	8250000
**3R**	27850000
**X**	250000
**X**	350000
**X**	450000
**X**	550000
**X**	650000
**X**	750000
**X**	850000
**X**	950000
**X**	1050000
**X**	1150000
**X**	1250000
**X**	1350000
**X**	1450000
**X**	1550000
**X**	1650000
**X**	1750000
**X**	1850000
**X**	1950000
**X**	2050000
**X**	2150000
**X**	2250000
**X**	2350000
**X**	7350000
**X**	11450000
**X**	14150000
**X**	16350000
**X**	16450000
**X**	16550000
**X**	16650000

Supplementary Table S2. 100 kb regions that consistently have values of heterozygosity less than 0.2 across all 5 B populations.

Chromosome	Start of region
**2L**	2505390
**2L**	22705390
**2L**	22805390
**2R**	19110083
**2R**	20310083
**3L**	3719855
**3L**	9419855
**3L**	9819855
**3L**	24019855
**3R**	14200284
**3R**	14300284
**X**	120552
**X**	220552
**X**	320552
**X**	420552
**X**	520552
**X**	620552
**X**	720552
**X**	820552
**X**	920552
**X**	1020552
**X**	1120552
**X**	1220552
**X**	1320552
**X**	1420552
**X**	1520552
**X**	1620552
**X**	1720552
**X**	1820552
**X**	1920552
**X**	2020552
**X**	2120552
**X**	2220552
**X**	2320552
**X**	3720552
**X**	14120552
**X**	14620552
**X**	16420552
**X**	16620552

Supplementary Table S3. Results from Pool-HMM being applied to 100kb windows samples from our neutral simulations. Shows the total number of instances where selection was detected across all 100kb regions taken from each set of neutral simulations.

Scenario	Migration Rate	Number of Simulated Populations	Total Number of Instances where Selection was Detected
**Neutral**	M=0	300	2
	M=1	300	2
	M=5	300	0
